# Recent Climatology (1991–2020) and Trends in Local Warm and Cold Season Extreme Temperature Days and Nights in Arabia

**DOI:** 10.3390/ijerph19052506

**Published:** 2022-02-22

**Authors:** Ali S. Alghamdi

**Affiliations:** Department of Geography, King Saud University, Riyadh 4545, Saudi Arabia; aorifi@ksu.edu.sa

**Keywords:** Arabian Peninsula, extreme temperature events, seasonal definition, acclimation range

## Abstract

The Arabian Peninsula (Arabia) is among the places to have experienced the greatest amount of warming during recent decades, and this trend is projected to continue. Specifics related to the characteristics (frequency, duration, and intensity) of extreme temperature events (ETEs) over Arabia as a whole are either largely outdated or limited only to specific areas. The seasonal ETE definitions commonly used in local studies are neither climatological- nor phenomenon-based. Using a novel and straightforward framework, the seasons of four extreme temperature types (extreme warm days/nights (EWDs/EWNs) and extreme cold days/nights (ECDs/ECNs)) were identified on the simultaneous basis of event occurrence and impact times. Assessments of ETE frequency, duration, and intensity and their recent changes were then provided based on the most recent climate data (1991–2020). Results showed that the use of traditional seasonal definitions (e.g., meteorological seasons) tends to assume a spatiotemporal homogeneity in the seasonality of ETEs and their potential risk levels throughout the year. The developed framework distinguished months with events that have larger potential impacts together with their local seasons. ETE seasons were found to vary at the regional and local scales and are better defined at both the local and phenomenon levels. Early extreme warm events were hotter, and those at locations with longer local warm seasons demonstrated higher intensities. ECDs tended to be more frequent at coastal locations, whereas ECNs were more frequent over southwestern Arabia. Early and late extreme cold events were much colder than those occurring mid-season. Trend analyses revealed generally increasing regional trends in the frequency of extreme warm events, whereas extreme cold events have declined. The duration (i.e., consecutive occurrences) and intensity of EWNs have been increasing at more locations, suggesting that urgent attention is needed within such an arid and hot climate type in which nighttime stress relief is already very limited.

## 1. Introduction 

The Arabian Peninsula (Arabia) is not only among the hottest places on Earth [1], but also among the places that have experienced the highest temperature increases during the last few decades [2]. For example, during the period 1901–2010, Attada et al. [3] found that the surface air temperature over Arabia increased at a higher rate than did the global mean surface air temperature across all seasons. Recently, Cook et al. [1] observed warming trends in the mean temperature over Arabia that were 1.4–2.1 times higher than those over the tropics and 2.3–3.1 times higher than global mean warming. Odnoletkova et al. [4] found that Saudi Arabia (approximately 80% of Arabia) experienced a 50% higher warming than that over land in the Northern Hemisphere during the last four decades. Almazroui [5] analyzed temperature changes over 11 subdomains across Africa and the Middle East and showed that under the RCP8.5 emissions scenarios, Arabia will not only warm at a faster rate, but will also experience higher variability compared with that of other subdomains. Studies have shown that the temperature over Arabia is expected to increase by more than 2 °C under any of the given scenarios by the end of this century (refer to Almazroui et al. [6] for references). It has been suggested that the absence of latent cooling over this dry region is the key reason for this pronounced warming over Arabia [1]. Given the ongoing and projected warming, the region is under a critical climate change threat that is affecting different sectors (e.g., human health, energy demands, and agriculture). For example, Pal and Eltahir [7] simulated the impacts of future climate change in the region and showed that the projected maximum temperature will be too high for human survival. 

One of the main consequences of the increasing temperature is that extreme hot events will become more intense and more frequent [8]. Extreme climate events are commonly used as indicators of climate change [9], as they present a greater threat to natural and human systems than does mean warming [8]. Studies on extreme weather events in Arabia have shown that temperatures have risen across the region particularly in the latter part of the 20th and early part of the 21st centuries (e.g., [10,11,12,13,14,15]). A common finding of these studies is that the frequency of extreme cold events has decreased, while that of extreme warm events has increased. However, the vast majority of these previous studies on temperature extremes were conducted in Saudi Arabia, and very little research has been conducted on the remainder of Arabia [14,16,17]. Most previous studies relied either on observational data prior to 2010 or a limited number of weather stations. Furthermore, researchers that focused on Saudi Arabia, covering approximately 80% of Arabia [13], assumed that the area would provide an indication regarding extreme temperatures over Arabia. However, Arabia has diverse spatial climatology [3,6], and trends in extreme temperature indices have been reported by most studies to exhibit different levels of magnitude and significance at various locations across Saudi Arabia and other parts of Arabia. For example, extreme temperatures over Saudi Arabia have shown different spatial patterns [18], and northern Arabia has experienced a more pronounced warming than southern Arabia [3]. Further, northern Arabia is projected to warm at a higher rate compared to southern Arabia [6,19]. 

Most studies related to warm days (WDs)/nights (WNs) and cold days (CDs)/nights (CNs) in Arabia have been based on the indices of the World Meteorological Organization Expert Team on Climate Change Detection and Indices (ETCCDI), and most of the analyses were performed at an annual or seasonal timescale with no monthly timescale analysis. The ETCCDI defines the WDs/WNs as the number (or percentage) of days with a maximum/minimum temperature higher/lower than the 90th/10th percentile. The results are commonly aggregated to the timescale of the analysis (i.e., annual or seasonal). In this type of analysis, extreme events are detected throughout the entire annual cycle. The analysis of these indices at an annual timescale may be more reasonable for the examination of changes in the mean climate and as indicators of climate change [9]. However, the impacts of extreme temperature events (ETEs) are not only sector-specific, but also time-specific [20]. ETEs at the upper (summer) and lower (winter) boundaries of the annual temperature scale tend to have more pronounced impacts across a number of different sectors. For example, extreme warm events during summer have significant effects across various sectors, including human health, the ecosystem, the economy, and agriculture [21]. Thus, studies tend to examine extreme warm/cold events for summer/winter months (e.g., [4,21,22]) or extended summer/winter seasons to account for early and late events (e.g., [10,20,23]). The meteorological season is typically used to define seasons; however, this definition assumes a spatial homogeneity in seasons (onset, end, and length), and it is not based on climate (e.g., [24,25]) or phenomena. Other studies have adjusted the meteorological seasons to account for climate change/warming (e.g., [26,27,28]).

ETCCDI indices have limitations, such as not capturing all aspects (frequency, duration, and intensity) of ETEs [20,23]. In fact, there has been no detailed analysis of the duration (i.e., consecutive occurrences) and intensity of WDs/WNs and CDs/CNs over Arabia as an entire region. Knowledge regarding different aspects of ETEs and any changes or variability is beneficial for many sectors, including health and the environment. For example, a comprehensive analysis and understanding of the temporal behaviors of temperature extremes is a critical component in the development of effective early warning systems [29], planning for resilient public health systems [30], and strategies for ETE mitigation and adaptation [21]. This study aims to update the knowledge on ETEs in Arabia using an extreme temperature seasonal definition that considers both spatial and temporal differences. More specifically, the objectives of this work are to (1) identify the seasons of extreme warm days (EWDs)/nights (EWNs) and extreme cold days (ECDs)/nights (ECNs) and (2) provide an assessment of their frequency, duration, and intensity based on the most recent 30-year period (1991–2020, i.e., the most recent climate). 

## 2. Data and Methods

### 2.1. Data and Quality Control

**Figure 1:**
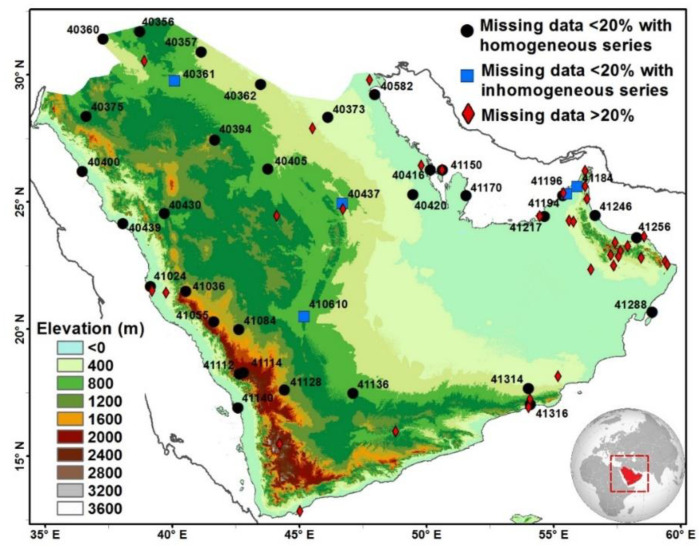
Map of the study area and weather stations along with their ID numbers.

Sub-daily temperature records for 1991–2020 were obtained from the Hadley Centre Integrated Surface Database (HadISD), available from the Met Office Hadley Centre [31]. HadISD is a quality-controlled dataset that was designed for extreme weather studies [32]. Thus, it was selected for this work. HadISD is a station-based dataset based on the Integrated Surface Database at the National Oceanic and Atmospheric Administration’s National Centre for Environmental Information. The dataset undergoes multiple quality control (QC) procedures, including diurnal cycle, unusual variance, and climatological outlier checks (refer to Dunn et al. [31]), with special attention paid to extreme values [32]. Before the analysis, the observation time was converted to the corresponding local time, as HadISD stores observation times in UTC. Daily maximum (TX) and minimum (TN) temperatures were calculated for days with eight (every 3 h) or more (e.g., every 2 h) observations. Stations with less than 20% flagged (missing) data from the study period (1991–2020) were used (e.g., [33,34]). These two QC steps resulted in the selection of 38 out of 70 stations (Figure 1).

### 2.2. Homogeneity

To examine the homogeneity of the station series, the penalized maximal F (PMF) test [35,36] was applied using the RHtestV5 package (a newer version of RHtestV4 [37]). This test was selected as it accounts for autocorrelation and type-I errors (detecting false breakpoints) equally distributed across points (dates) in the time series, unlike the standard normal homogeneity test [35,36]. The analysis was applied on the monthly series (e.g., [38]), as recommended by Wang and Feng [37]. The PMF test is an absolute statistical homogenization test that requires no reference series, with the null hypothesis that the time series under consideration is homogeneous. To minimize the probability of detecting false breakpoints (type-I errors), the PMF test was applied at the 99% confidence level (e.g., [33]), as no metadata were available. Five stations were detected to have significant inhomogeneous series (Figure 1).

For these stations, the penalized maximal *t* (PMT) test [35] can be applied to reexamine the homogeneity based on a homogeneous reference series. The PMT is a relative statistical homogenization test that requires a reference series; thus, the test can only be applied when a candidate station (i.e., the station to be homogenized) has a useful reference series. A reference station or weighted reference series is usually selected based on small elevation differences, short distances, and high correlation [39,40]. Distance and height difference criteria are critical, as high correlations between reference and candidate stations do not necessarily indicate similarity in the regional climatology [40]. Based on the criteria of a distance of <300 km, a correlation coefficient of 0.95 [39], and a minimum of three neighboring stations [41], none of the candidate stations had a useful reference series. Accordingly, all these five stations were excluded, and the data from the remaining 33 stations were used (Figure 1 and Table S1). Previous studies on ETEs over Arabia were based on smaller numbers of stations (19 stations in [16], 13 stations in [17], and 23 stations in [14]).

### 2.3. Thermal Extreme Indices and Extreme Season Definitions

A WD (or WN) event was defined as a day (or night) with a TX (TN) equal to or higher than the 90th percentile (Table 1). Similarly, a CD (or CN) event was defined as a day (night) with a TX (TN) equal to or lower than the 10th percentile (Table 1). These percentile thresholds were selected to optimize the balance of extreme versus other temperature events [20], and to account for the nature of the temperature regime of Arabia (arid and hot environment). The percentile technique was used to account for different local climates and was estimated from the base period of 1991–2020 (the most recent climate) on a 15-day-centered window to account for seasonal and monthly cycles (e.g., [20]). If percentiles are estimated on an annual or seasonal basis, the colder (warmer) months will dominate the determination of the cold (warm) events (e.g., [23]). A calendar-day percentile is a time-relative threshold, by which it is possible to detect warm events during cold months and cold events during warm months (e.g., [20]). However, this timing aspect is more an occurrence-relative measure than a severity/impact-relative measure that can account for the level of potential impacts relative to the time of the year according to long-term acclimatization adjustment. Naturally, humans and other species are adapted to their local environment, as they develop a climatological acclimatization adjustment, which defines their acclimation/tolerance range. For instance, plants have the ability to develop a thermal tolerance range and can adapt to extreme seasonal anomalies within that range [42]. Thus, a plant can adapt and survive a relatively warm day during winter as long as the event has a temperature within the acclimation range (i.e., climatological acclimatization). Studies on the health impacts of ETEs have shown that the magnitude and risks imposed by ETEs vary temporally not only between seasons, but within seasons, i.e., from month to month (e.g., [43,44]).

To simultaneously account for the occurrence and impact times (i.e., thermal tolerance) of events and to delineate seasons of different ETEs that have higher potential impacts (i.e., warm and cold seasons), a straightforward ETE-based framework was developed in two steps. First, the climatological distributions of TX and TN of the detected WDs/WNs and CDs/CNs were computed for each ETE type. Then, WDs and WNs with TX and TN greater than the corresponding 75th percentile (WDT_75th_/WNT_75th_) were recognized as extreme warm events (EWDs/EWNs). Similarly, CDs and CNs with TX and TN below the corresponding 25th percentile (CDT_25th_/CNT_25th_) were obtained and identified as extreme cold events (ECDs/ECNs). Thus, a relatively WD or WN during winter, or even summer, was not counted as an extreme event (i.e., EWD or EWN) unless the observed temperature (TX/TN) was well above the climatological acclimatization and adaptation capability (i.e., thermal tolerance range). The TX and TN of warm and cold days/nights can be thought of as an absolute intensity index, which can be used to infer event magnitudes and impacts and, thus, the climatological tolerance range. The lower and upper quartiles (25th and 75th percentiles) were considered because the distributions from which both thresholds were defined were already distributions of cold and warm events. Both thresholds would also allow the consideration of early and late events as sudden changes in the thermal conditions that could result in substantial impacts [45]. To account for spatial variability, the framework was applied at the station level.

Event intensities (warm and cold) were defined based on relative indices, as relative indices would allow to the comparison of events not only across different months, but also across different indices (warm/cold days vs. warm/cold nights) and different locations (Table 1). All indices applied were calculated using the mean statistics instead of using the maxima statistics (e.g., the highest temperature or the longest duration). Analyzing trends in indices that are maxima-based could lead to biased results, as such rare intense/long events at the beginning (end) of the series may result in decreasing (increasing) trends. Further, rare extreme events would omit frequent medium-sized events, and the mean climatology would not be fully captured. For example, using the length of the longest events would be more an indicator of the upper limits of event duration [23] rather than of the mean climatology.

### 2.4. Trend Analysis

Using Sen’s [46] slope estimator with the Mann–Kendall test, temporal trends in the components (frequency, intensity, and duration) of ETEs were estimated. Following Yue et al. [47], a time series was pre-whitened when a serial correlation was detected. It is well known that the power of the Mann–Kendall test is affected by the presence of serial correlation in a time series. Significances were assessed at the 10% level. To account for spatial variability in event characteristics, both absolute trends and relative changes were examined [21]. Absolute trends were transformed into relative changes (%) with respect to the averages of each index (1991–2020).

## 3. Results and Discussion

### 3.1. Evaluation

**Figure 2:**
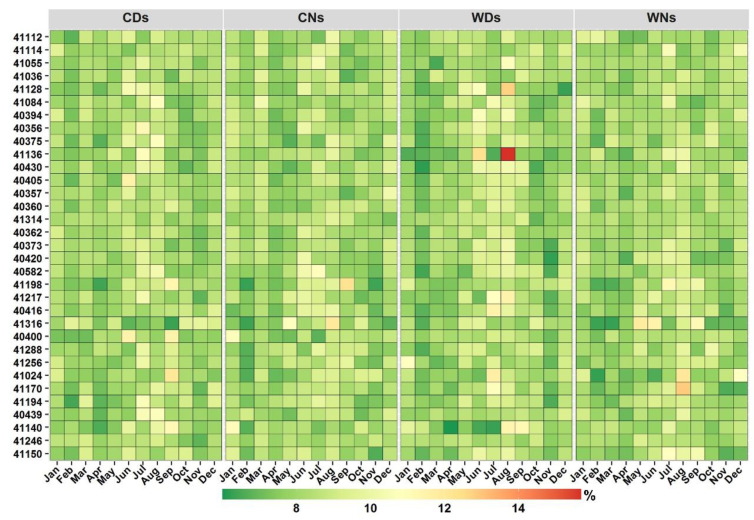
Monthly proportion of annual mean frequency for cold days (CDs), cold nights (CNs), warm days (WDs), and warm nights (WNs). Stations (*y*-axis) are ordered from high to low elevation. Refer to Figure S1 for spatial patterns.

To evaluate the proposed method, the general monthly spatiotemporal patterns of the average frequency of CDs, CNs, WDs, and WNs are presented first (Figure 2). A general analysis of these events based on calendar-day percentile is essential to establish a baseline for evaluating the proposed framework. Although there is a notable spatial variability (Figure S1), the temporal variabilities at the station level are low in most of the indices (Figure 2). A number of stations (e.g., 41314 and 41246) showed almost comparable monthly averages, and the majority of stations showed relatively constant values. Only a few indices at a few stations demonstrated distinct monthly differences (e.g., 41140 and 41217). Interestingly a number of stations exhibited frequent cold events during warm months (e.g., 41316, 40400, and 41024), whereas other stations displayed higher averages of both warm and cold events in the same months (e.g., 41140). When looking at the monthly event contribution to the total events, there are almost no significant differences across most of the stations (Figure 2). Such a low level of temporal differences is due in part to the use of the calendar-day percentile method to detect events. From a climatological perspective, this could indicate a lack of seasonal cycles or high year-to-year variabilities (either in the mean or variance). The former is less relevant to Arabia, as Arabia has a clear temperature seasonal cycle given its climate type (low-latitude warm desert (BWh) and low-latitude semi-arid steppe (BSh), according to the Köppen climate classification).

Arid environments have a pronounced interannual variability, and it was noted that a number of the cold (warm) days/nights detected during summer (winter) were in mild summer (winter) months (Figure S2). This highlights the importance of distinguishing months and seasons with events that have higher potential impacts and, also, suggests the importance of variabilities in the mean climate. To obtain further insights, the associations between anomalies in the mean and variance of TX and TN and anomalies in the occurrence of warm and cold events were explored at a monthly timescale (Figure S3). Anomalies were calculated to account for spatial and temporal variabilities; thus, a direct comparison can be made. Warm and cold events correlated most strongly with variability in the means, followed by variability in the variances. This would suggest that the occurrences of monthly warm and cold events were more closely related to the variability in the mean climate, which is consistent with previous studies (e.g., [48,49,50,51]).

**Figure 3:**
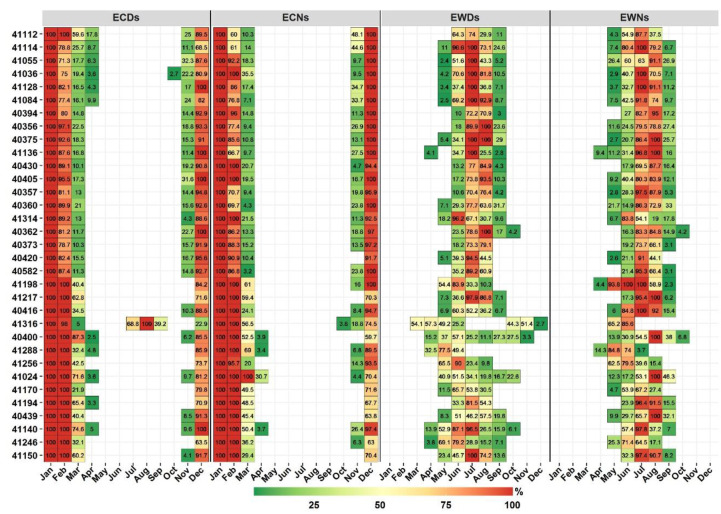
Monthly occurrence percentages of ECDs, ECNs, EWDs, and EWNs. Stations (*y*-axis) are ordered from high to low elevation.

**Figure 4:**
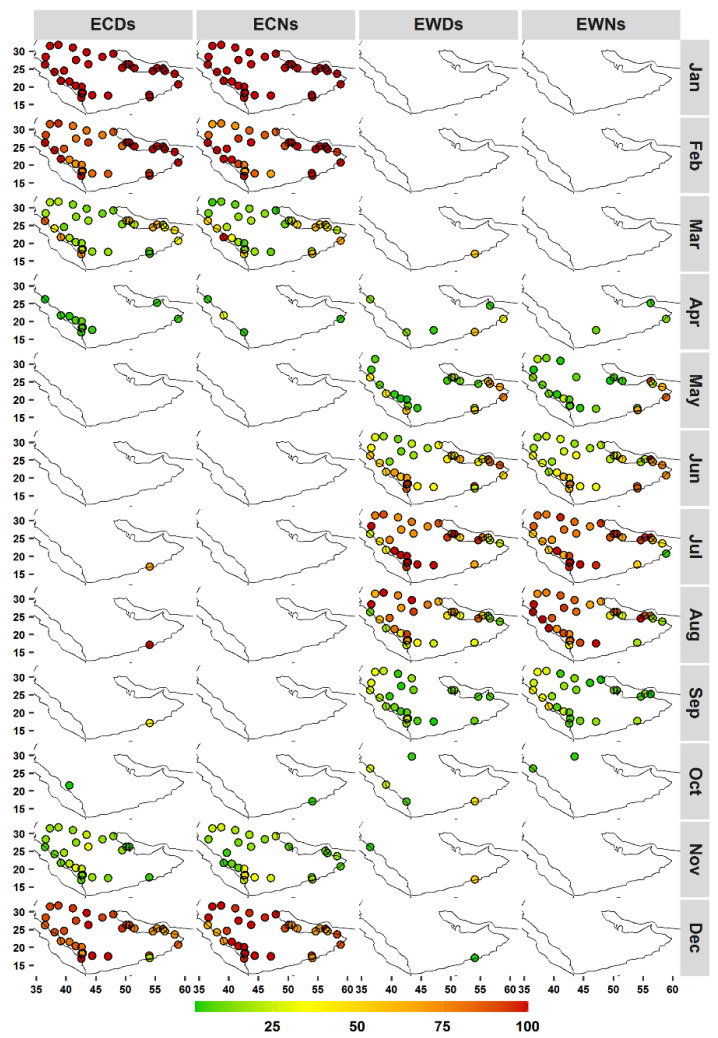
As in Figure 3, but showing the spatial distributions of indices by month.

These results (Figure 2, Figures S1 and S2) are consistent with the common use of such temperature extreme indices as indicators of changes/variability in the mean climate (e.g., [9]). However, the observed monthly patterns highlight the importance of distinguishing months and seasons with events that have higher potential impacts. Figure 3 shows the results of the developed framework, in which events are classified into two categories based on their absolute temperatures (i.e., TN and TX) using the corresponding 25th and 75th percentiles (WDT_75th_/WNT_75th_ and CDT_25th_/CNT_25th_). Almost all detected CNs and CDs from Apr to Oct had TNs higher than the CNT_25th_ and TXs higher than the CDT_25th_ at most stations. A large portion of Dec to Feb CDs and CNs had TNs < CDT_25th_ and TXs < CNT_25th_. This would indicate that cold events detected between Dec to Feb (Apr to Oct) were characterized by cold (normal) temperatures and were beyond (within) the acclimatization and adaptation capability. Mar and Nov can be considered to be transitional months for cold events at a large number of stations. However, there is a clear geographical variability in these months, as extreme cold events tend to constitute notable fractions of the Mar cold events at stations at lower altitudes (Figure 3 and Figure 4). During Apr, five stations, at higher altitudes, experienced few ECDs, and three stations along the west coast experienced few ECNs. Station 41316 deviated from these patterns as 100%, 98%, 5%, 69%, 100%, 39%, and 23% of the detected CDs in January, February, March, July, August, September, and December, respectively, had TXs below the CDT_25th_ (Figure 3). This distinctive pattern is related to the unique temperature distribution due to the influence of the monsoon season in May–September (Figure S4).

For warm events (WDs and WNs), there was a higher spatiotemporal variability compared with that for cold events (Figure 3 and Figure 4). Warm events during October to April had temperatures (TX and TN) below the WDT_75th_ and WNT_75th_ at most of the stations. However, four stations had no EWDs in May. July and August tended to be the months in which a large number of stations had all or large portions of their WDs/WNs classified as extreme warm events (TX/TN > WDT_75th_/WNT_75th_). In June, a large portion of WDs were classified as EWDs at most stations, except at northern stations. Four stations located in the southeast and three additional stations along the west coast had 50% or more of their May WNs exhibiting temperatures higher than WNT_75th_. These stations showed a similar pattern in the WN index. Station 41316 also deviated from the other stations and experienced 54% (March), 88% (April), 50% (May), 26% (June), 45% (October), 52% (November), and 3% (October) of its WDs with higher temperatures (>WDT_75th_) (Figure 3). Station 41288 showed relatively similar patterns, and no extreme warm events were detected in Aug and Sep. As already noted for station 41316, the seasonal temperature cycle of station 41288 had a binomial distribution (Figure S4). However, station 41288 had lower temperature variabilities compared with that at station 41316, which may explain the absence of ECDs and ECNs during warm months at station 41288.

### 3.2. Implications

Overall, Figure 2 shows no clear monthly variations in the mean frequency of warm and cold events at most stations. Due to the use of the calendar-day percentile, which allowed events to be detected throughout the year, and high year-to-year variability, numerous warm (cold) events that were detected during winter (summer) were in months with relatively mild (less warm) winters (summers). Such events do not necessarily have major negative impacts, as they fall within the long-term (climatological) acclimation/tolerance range, and may be considered to be a period of stress relief. This is supported by results from Figure 3 and Figure 4, as most of these off-meteorological-season events were not characterized by extreme temperatures. This would indicate the importance of simultaneously considering the timing of both the occurrence and impact of events. It is important to note that the proposed framework was still detecting ETE during seasons of extremes (winter and summer). However, the established framework was able to account for both aspects of timing (occurrence and impacts) and delineate ETE seasons with useful spatial and temporal results. For example, the framework showed that the season of extreme warm events was from Jun to Aug at most stations. However, it detected some spatial variability at regional and subregional scales. For example, the northwest stations had lower percentages of extreme warm events (EWDs and EWNs) in Jun and a higher frequency in Sep compared with that at other stations. This indicates that the core season of extreme warm events at northwest stations starts later (Jun) and lasts longer (until Sep). However, extreme warm events occur earlier (Apr) at southeastern stations. At the subregional scale, station 41055, for example, experienced earlier and higher percentages of EWNs in May and Sep compared with that at other stations at higher elevations in southwest Arabia. It was noted that the TN distribution at this station was characterized by two small peaks in these months. 

Consequently, extreme seasons are better defined at both local (i.e., at individual stations) and phenomenon (i.e., individual extreme temperature indices) levels, as the use of the traditional seasonal definition (e.g., meteorological seasons) would assume spatiotemporal homogeneity. The framework revealed that using the meteorological seasons, or even the related extended seasonal approach, would not account for the spatial variability in the seasonality of different ETEs. For instance, at four stations, all May WDs had TXs below their WDT_75th_ (Figure 3), and thus May should not be counted as a summer or warm-season month at these stations when dealing with warm temperature events. Similarly, Apr and Oct are not commonly considered to be warm-season months; however, a number of WDs at nine stations were found to have TXs above the WDT_75th_ (Figure 3). Further, seven (eight) stations did not experience any ECNs (ECDs) in Nov, which is commonly considered to be in a cold/winter season (Figure 3). Although the percentages of EWDs were not high at some of these stations, early events are still of importance as they could have major health impacts [45]. Even when the WDT_75th_/WNT_75th_ (CDT_25th_/CNT_25th_) thresholds were increased (reduced) to the 85th (15th) percentile, a number of stations were found to still experience extreme warm (cold) events during these transitional months. It is also important to account for these events given the ongoing regional changes in the mean climate (e.g., warming, variability, and sensitivity). In fact, a number of early and late events were found to have higher intensities (Section 3.3). Hence, a local season definition based on the magnitude and type of events (WDs vs. WNs) is rational and would improve representations and implications.

Because this framework was based on the absolute temperatures (TX and TN) of events, it is reasonable to ask whether the detected extreme cold/warm events (ECEs/EWEs) had lower/higher relative intensities (deviations from the corresponding percentiles) compared with that of non-ECEs/EWEs. The differences between the average relative intensities of ECEs/EWEs and those of non-ECEs/EWEs were calculated, and all ECEs/EWEs had high relative intensities across all stations and indices (Figure S5). This indicates that ECEs/EWEs are extreme in both absolute and relative senses, which supports the developed framework and the use of lower and upper quintiles (25th and 75th percentiles). The identified patterns of ECEs/EWEs at stations 41316 and 41288 further suggest the suitability of the framework when adjusting for different seasonalities in temperature distribution. Accordingly, extended local warm and cold seasons were defined at the station level using the proposed framework to examine and document the climatology (frequency, duration, and intensity aspects) of extreme warm/cold days and nights. An extended season approach was considered to account for early and late events that may have significant impacts.

### 3.3. Extreme Warm Day and Night Behavior

**Figure 5:**
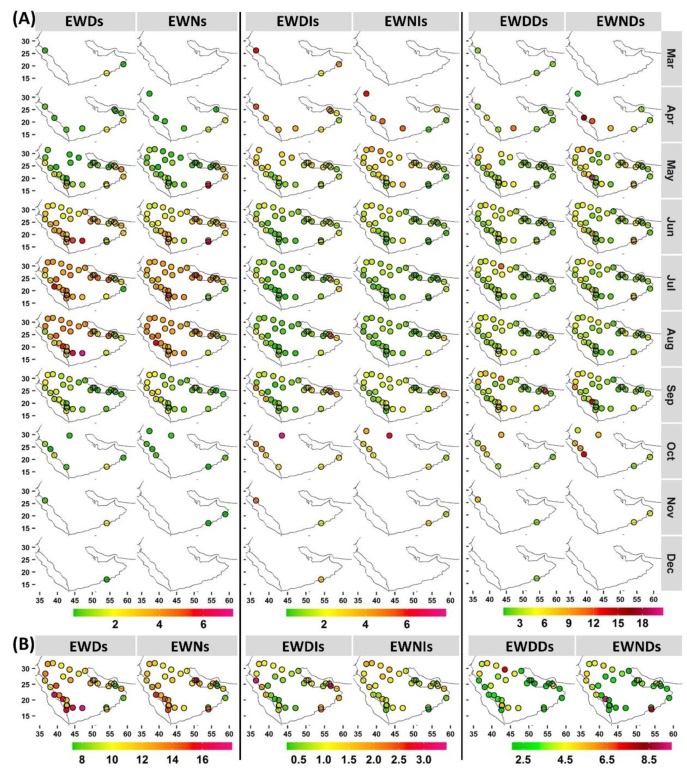
(**A**) Monthly mean frequency (**left**), intensity (**middle**), and duration (**right**) of extreme warm days and nights, along with corresponding (**B**) local seasonal averages. For exact values, refer to Figure S6.

The occurrence of extreme warm events (EWDs and EWNs) increased as the local warm season progressed at most of the stations (Figure 5). Coastal stations tended to experience earlier (Apr) EWDs and EWNs. Further, late warm events (Oct) were also more common at these stations. This may indicate the important role of land–sea interactions (e.g., sea breeze). In May, EWNs were more common at a large number of stations than EWDs. Jul and Aug can be considered to be the regional warm season core in which most of the stations recorded their highest EWD and EWN mean frequencies. However, southeastern stations experienced their highest EWD and EWN mean frequencies earlier (May and Jun). Although most stations showed a lower mean frequency of extreme warm events (~1.2 day/night) in Sep, stations in northwest Arabia displayed a higher mean frequency (~2 days/nights). At the local warm season scale, southwestern stations showed higher mean values (~16 events, on average), followed by stations in northern Arabia (~12 events, on average). Nevertheless, EWDs and EWNs at most of the southwestern stations (at higher elevations) were characterized by lower intensities (EWDI and EWNI). In contrast, stations in northern Arabia tended the most to display higher intensity and duration mean values. This would suggest differences in the importance of regional and local factors (e.g., elevation, aridity, land cover, distance from bodies of water, and atmospheric circulation) in determining different aspects of events (frequency, duration, and intensity). For example, Alghamdi and Harrington [52] suggested that the spatial patterns of heat wave intensities in Saudi Arabia are more related to local factors.

Intensity indices (EWDI and EWNI) indicated that early events were characterized by higher relative intensities (>3 °C) than mid-season events at most of the stations (Figure 5). During the local mid-warm-season, most stations had mean intensity values hovering around 1.5 °C. Extreme warm events at stations with longer local warm seasons demonstrated a higher intensity (3.5–7 °C). To some extent, the duration indices displayed similar patterns at most of the stations, which tended to experience events with a mean duration of approximately 3 days/nights. Longer events were more common at northern stations. These results indicate the importance of early and late events, even when they have a low rate of occurrence. At the warm season scale, coastal stations tended to experience warmer EWDs, followed by stations farther north. For the former, this indicates higher levels of stress during the daytime, given the presence of more moist air. However, EWNs were warmer at northern stations, and coastal stations tended to show lower EWNI values. 

### 3.4. Extreme Cold Day and Night Behavior

**Figure 6:**
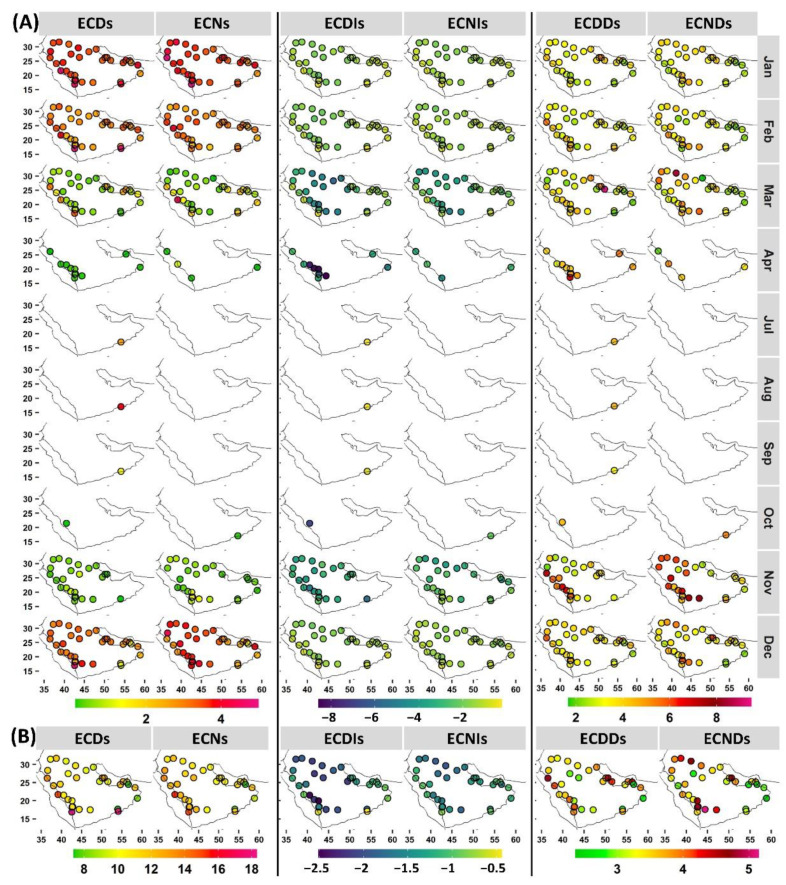
(**A**) Monthly mean frequency (**left**), intensity (**middle**), and duration (**right**) of extreme cold days and nights, along with corresponding (**B**) local seasonal averages. For exact values, refer to Figure S6.

Extreme cold events (ECDs and ECNs) exhibited less spatiotemporal variability compared with that of extreme warm events (Figure 6). Nov was the start of local ECD and ECN seasons at almost all stations (except at stations 41036 and 41316, where it was Oct) with a mean frequency of approximately 0.8 (0.4) events (Figure 6). Jan was the peak month of ECDs and ECNs at almost all stations, with a mean occurrence of four to five events and a mean duration of approximately 2.5 days/nights. Although Mar is the end month of extreme cold events at a large number of stations, western stations, mostly over the southwestern region, along with two other stations (41194 and 41288), continued to experience ECDs in Apr. Four of these stations also experienced ECNs in Apr. Interestingly, a number of these stations still experienced warm events in this month. This would indicate a high variability of the daily temperature in Apr. These stations are along the coastlines, which highlights the importance of land—sea interactions. At the local cold season scale, higher mean ECD numbers tended to occur at coastal stations, whereas higher mean ECN numbers occurred largely at southwestern stations. Early and late extreme cold events had more intense temperatures compared with those in the local mid-season (Figure 6). Generally, ECDs were more intense than ECNs. While stations in southwest Arabia recorded the most intense ECDs followed by ECNs, northern stations recorded the most intense ECNs, followed by ECDs. Extreme cold events at coastal stations were characterized by lower intensities. The duration aspect did not show high temporal variabilities at the monthly scale, except in Nov, in which longer events were more common (Figure 6). This highlights the importance of early events.

### 3.5. Trends

**Figure 7:**
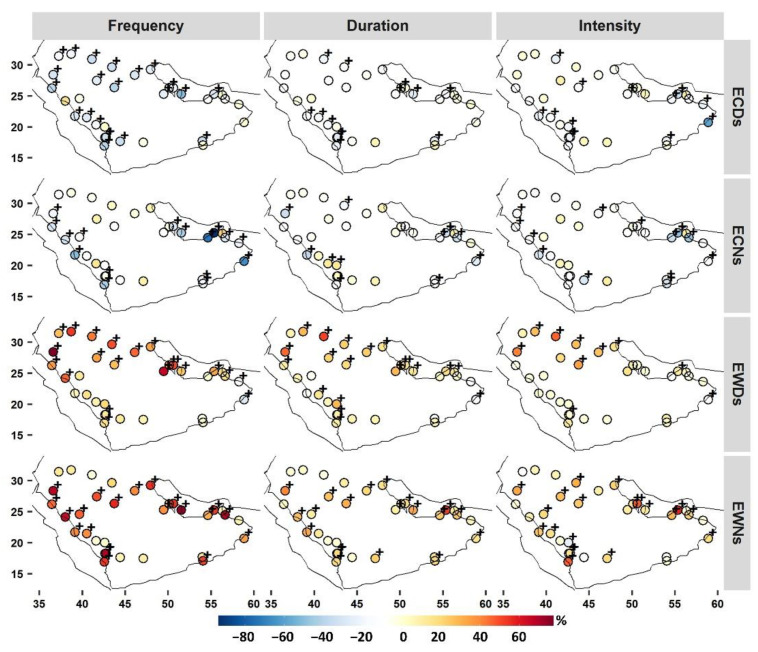
Relative temporal decadal trends in frequency, duration, and intensity aspects of ETEs during local seasons. +α = 0.10 level of significance.

Trend analyses were applied at the local seasonal time scale for each index. An overall analysis revealed regional upward trends in the frequency of extreme warm events (EWDs and EWNs), whereas the trends for extreme cold events (ECDs and ECNs) declined (Figure 7 and Figure S8). This is in line with observed trends in TX and TN (Figure S7), and consistent with the results of previous local research (e.g., [13,14,15,17]). Although previous local studies have used different methods and data, the agreement here confirms the ongoing changes in ETEs in the region. Relative changes in the frequency indices tended to be much more pronounced (Figure 7). EWNs tended to experience higher relative warming changes in both frequency and intensity indices (Figure 7). EWNs increased at almost all stations, and this change was statistically significant at 21 stations with rates ranging from 3 to 9 nights/y^−1^ (which translates to 33–78% relative trends/year^−1^). Nineteen of these stations, along with an additional station, experienced significant increasing trends in their EWND (0.7–2 nights/year^−1^), and coastal stations tended to exhibit more pronounced rates of change. For EWNI (relative intensity), significant warming trends were detected at nineteen stations (0.1–0.5 °C/year^−1^), fifteen of which experienced significant increasing trends in their EWNs and EWND. Although four stations showed decreasing trends in EWNI, only one station (41084) showed a significant trend of −0.2 °C/year^−1^ (−23%/year^−1^). 

The frequency of EWDs showed significant increasing trends at 19 stations (0.5–10.5 days/yr^−1^, or 17–77%/yr^−1^). Most of these stations are clustered along the east coast and in northern Arabia. However, two stations (41288 and 41256) showed significant decreasing trends in their EWDs. These two stations, located in southeastern Arabia, showed trends of −2.2 days/yr^−1^ (−27.50%/yr^−1^) and −2.1 days/yr^−1^ (−17%/yr^−1^), respectively, and experienced no significant trends in their local EWD season TX (Figure S7). The insignificant increasing trends in EWDs were clustered mostly at highland stations. Trends in the EWDD index showed relatively similar spatial patterns with significant increasing trends ranging from 0.4 to 2.4 days/yr^−1^ (10% to 52%/yr^−1^), with higher rates in northern Arabia. Five stations showed decreasing trends in EWDD, only one of which (41288) showed a significant trend of −0.4 days/yr^−1^ (18%/yr^−1^). On the contrary, significant warming trends in the EWDI (0.1–0.5 °C/yr^−1^) were detected at nine stations, mostly over northern Arabia.

For ECDs, significant decreasing trends were detected at 22 stations with slopes of 1.8–7.2 days/yr^−1^ (18–55%/yr^−1^). The remaining stations, mostly along the southeastern coastal areas, showed either insignificant decreasing trends or insignificant increasing trends. Although the ECDD index showed decreasing trends across much of Arabia, significant trends were found at 13 stations. These stations were mostly located in highland areas and along the eastern coast. The majority of stations (85%) showed warming signs in their ECDI index, which were significant (0.2–0.5 °C/yr^−1^) at 13 stations. Most inland stations did not experience significant trends in their ECNs. Significant decreasing trends in ECDs were found at 16 stations, largely in coastal areas, with rates ranging from −1.7 to −10 nights/yr^−1^ (17% to 93%/yr^−1^). The ECND displayed decreasing trends at a large proportion of the stations, with significant trends at only nine stations. However, two stations (41055 and 41084) showed increasing trends in their ECND, with rates of 0.4 and 0.6 days/yr^−1^ (11% and 13%/yr^−1^), respectively. ECNs have become less cold at 13 stations, as these stations showed significant increasing trends in their ECNI indices.

## 4. Summary and Conclusions

Using homogeneous and quality-controlled data from 33 stations, the monthly temporal and spatial patterns in four extreme temperature types (WDs, WNs, CDs, and CNs) across Arabia were explored for the most recent climate period (1991–2020). Despite the observed notable spatial differences in these indicators, monthly variabilities at the station level were low. This was related to the use of the calendar-day percentile method and the interannual variability in the mean temperatures. This threshold approach assumed that events had similar levels of risk throughout the year, as it did not consider acclimation/tolerance range. To account for potential impacts, this approach was further extended by incorporating the absolute intensity of events (i.e., TX and TN). This framework distinguished months with events that had higher potential impacts, together with their local seasons. The framework showed that extreme temperature seasons varied at regional and local scales, and that seasons are better defined at both local (i.e., at individual station) and phenomenon (i.e., extreme temperature type) levels. It was noted that the use of the traditional seasonal definition (e.g., meteorological seasons) assumes spatiotemporal homogeneity and would not account for the spatial variability in ETE seasonality.

Extreme warm events (EWDs and EWNs) increased as the local warm season progressed at most of the stations (Figure 5). Coastal stations tended to experience earlier EWDs and EWNs, and southwestern stations showed higher frequencies, followed by stations in northern Arabia. The intensity index indicated that early events were characterized by higher relative intensities, and extreme warm events at stations with longer local warm seasons tended to be warmer. Most stations tended to experience events with mean durations of approximately 3 days/nights, and longer events were more common over northern Arabia. For extreme cold events (ECDs and ECNs), Nov was the start of the local season at almost all stations. Mar was the end month of extreme cold events at a large number of stations. However, ECDs lasted until Apr at western stations, mostly in southwestern areas. Higher mean ECDs tended to occur at coastal stations, whereas higher mean ECNs were largely located at southwestern stations. Early and late extreme cold events had more intense temperatures compared with that of local mid-season events, and ECDs were more intense than ECNs in general. Stations in southwestern Arabia recorded the most intense ECDs, followed by ECNs, whereas northern stations recorded the most intense ECNs, followed by ECDs. 

Trend analyses revealed general regional increasing trends in the frequency of extreme warm events (EWDs and EWNs), whereas the trends for the frequency of extreme cold events (ECDs and ECNs) decreased. EWNs and EWDs became significantly longer at 61% and 58% of the examined locations, respectively, and coastal stations (and over northern Arabia for EWDs) tended to exhibit more pronounced rates of change. EWNs and EWDs became significantly warmer at 58% and 27% of stations, respectively. This suggests that EWNs should be of more concern for adaptation and response planning for different sectors (e.g., health and environment). Within such an arid and hot climate type, the daytime warm season temperature is already high, and thus nighttime stress relief, which is limited, is very necessary [23]. 

This study establishes and illustrates the importance of defining extreme temperature seasons relative to their local occurrence and potential impacts. Early warning systems are not available for some of the countries within Arabia, and this study not only calls for their establishment, but also suggests how they can be activated at a local scale for better outcomes considering the observed variability in the local seasonalities of extreme warm and cold seasons. Studies have shown that early warning systems are better implemented at the local scale to improve the accuracy of the warning and emergency preparedness [53]. This developed framework can help in such an important effort, as it provides a straightforward scheme not only to distinguish extreme temperature events of possible great risk but also to recognize their seasons. The framework could be simply adapted for different applications and at different places by adjusting the percentile threshold (i.e., WDT/WNT_75th_ and CDT/CNT_25th_). For example, the upper (lower) percentile threshold could be lowered (increased) for high latitude climates to account for the higher (lower) sensitivity to extreme warm (cold) events. Such an adjustment could also be made for agriculture and energy sector applications. 

However, this framework was developed from a climatological perspective. Population vulnerability and acclimatization processes vary through time, which imposes a considerable level of uncertainty due to the unscaled and complex association between acclimatization and extreme effects [23]. Incorporating this aspect when defining temperature extremes and their seasonality would improve the preparedness of society for the future challenges and effects of climate change. Research on risk and vulnerability assessments related to climate extremes in this region are limited, and future work on these topics is encouraged. 

## Figures and Tables

**Table 1:** Definitions of extreme temperature indices developed and used in this study. TX: daily maximum temperature; TN: daily minimum temperature.

No.	Indices	Name	Definition	Units
1	WD	Warm days	Days with TX ≥ 90th percentile.	Days
2	EWD	Extreme warm day frequency	Monthly number of events in which WD experiences TX ≥ 75th percentile of TX of WDs (WDT_75th_).	Days
3	EWDD	EWD duration	Mean length of consecutive occurrences of each EWD.	Days
4	EWDI	EWD intensity	Average difference between TX of each EWD and TX_90th_.	°C
5	WN	Warm nights	Nights with TN > 90th percentile.	Days
6	EWN	Extreme warm nights frequency	Monthly number of events in which WN experiences TN ≥ 75th percentile of TN of WNs (WNT_75th_).	Days
7	EWND	EWN duration	Mean length of consecutive occurrences of each EWN.	Days
8	EWNI	EWN intensity	Average difference between TN of each EWN and TN_90th_.	°C
9	CD	Cold days	Days with TX ≤ 10th percentile.	Days
10	ECD	Extreme cold days frequency	Monthly number of events in which CD experiences TX ≤ 25th percentile of TX of WDs (CDT_25th_).	Days
11	ECDD	ECD duration	Mean length of consecutive occurrences of each ECD.	Days
12	ECDI	ECD intensity	Average difference between TX of each ECD and TX_10th_.	°C
13	CN	Cold nights	Nights with TN ≤ 10th percentile.	Nights
14	ECNF	Extreme cold nights frequency	Monthly number of events in which CN experiences TN ≤ 25th percentile of TN of CNs (CNT_25th_).	Nights
15	ECND	ECN duration	Mean length of consecutive occurrences of each ECN.	Nights
16	ECNI	ECN intensity	Average difference between and TN of each ECN and TN_10th_.	°C

## Data Availability

Publicly available datasets were analyzed in this study. This data can be found here: https://www.metoffice.gov.uk/hadobs/index.html, accessed on 13 February 2022.

## Supplementary Materials

The following supporting information can be downloaded at: https://www.mdpi.com/article/10.3390/ijerph19052506/s1, Table S1: Weather stations with World Meteorological Organization (WMO) ID, latitude, longitude, and elevation. Stations are ordered from high to low elevation; Figure S1: Mean frequency of cold days (CDs), cold nights (CNs), warm days (WDs), and warm nights (WNs) by month; Figure S2: Annual cycles of daily maximum temperature [TX] (black points, °C) at station 40430 along with 90th (red line), 10th (blue line) percentiles, monthly mean TX (green line), detected WDs (red points), and CDs (purple points). Percentiles are estimated from the base period of 1991–2020 on a 15-day centered window; Figure S3. Correlation coefficients (Spearman’s rank) between anomalies of mean/variance of TX (TXm/TXv) and TN (TNm/TNv), and frequency of different temperature extreme types. + is statistically significant at the 90% level. All anomalies were obtained with respect to the 1991 to 2020 monthly climatological average. Stations (*y*-axis) are ordered from high to low elevation; Figure S4. Annual cycles of maximum (red) and minimum (blue) temperatures (1991–2020) at stations 412888 and 41316; Figure S5. Differences between average relative intensities (°C) of extreme warm/cold days/nights [EWDs/ECDs, ECDs/ECNs] and warm/cold days/nights [WDs/CDs, WNs/CNs] by months. Stations (*y*-axis) are ordered from high to low elevation; Figure S6. Monthly and seasonal means of frequency, intensity, and duration of extreme warm/cold days and nights along with the corresponding local seasonal averages. Stations (*y*-axis) are ordered from high to low elevation; Figure S7. Absolute temporal decadal trends in mean daily maximum (TXm) and minimum (TNm) temperatures at local seasons of each ETEs. + is statistically significant at the 90% level. Stations (*y*-axis) are ordered from high to low elevation; Figure S8: Absolute temporal decadal trends in frequency, duration, and intensity aspects of ETEs during local seasons. + α = 0.10 level of significance.
