# Supplementary material for: Recent Climatology (1991–2020) and Trends in Local Warm and Cold Season Extreme Temperature Days and Nights in Arabia
Source: Int J Environ Res Public Health. 2022 Feb 22;19(5):2506. doi: 10.3390/ijerph19052506 (PMC8909031; doi:10.3390/ijerph19052506)
Supplement: Supplementary file 1 [file ijerph-19-02506-s001.zip › ijerph-1565738-supplementary.pdf]

Supplementary material

Table S1. Weather stations with World Meteorological Organization (WMO) ID, latitude, longitude, and elevation. Stations are ordered from high to low elevation.

No.	WMO ID	Lon (°E)	Lat (°N)	Elevation (m)	Name
1	41112	42.657	18.24	2090.3	ABHA
2	41114	42.804	18.297	2065.9	KING KHALED AB
3	41055	41.634	20.296	1672.1	AL BAHA
4	41036	40.544	21.483	1477.7	TAIF
5	41128	44.419	17.611	1213.7	NEJRAN
6	41084	42.621	19.984	1184.8	BISHA
7	40394	41.686	27.438	1015.3	HAIL
8	40356	38.731	31.693	854.4	TURAIF
9	40375	36.619	28.365	777.5	TABUK
10	41136	47.121	17.467	720.2	SHARURAH
11	40430	39.705	24.553	655.6	PRINCE MOHAMMAD BIN ABDULAZIZ
12	40405	43.767	26.3	648	GASSIM
13	40357	41.138	30.907	552.6	ARAR
14	40360	37.279	31.412	509.6	GURIAT
15	41314	54.025	17.666	478.5	THUMRAIT
16	40362	43.491	29.626	449.3	RAFHA
17	40373	46.125	28.335	357.8	QAISUMAH
18	40420	49.485	25.285	179.2	AL AHSA
19	40582	47.969	29.227	62.8	KUWAIT INTL
20	41198	56.324	25.112	46.3	FUJAIRAH INTL
21	41217	54.651	24.433	26.8	ABU DHABI INTL
22	40416	50.152	26.265	25.6	KING ABDULAZIZ AB
23	41316	54.091	17.039	22.3	SALALAH
24	40400	36.476	26.199	20.1	WEJH
25	41288	58.89	20.675	19.5	MASIRAH
26	41024	39.157	21.68	14.6	KING ABDULAZIZ INTL
27	41256	58.284	23.593	14.6	SEEB INTL
28	41170	51.565	25.261	10.7	DOHA INTL
29	41194	55.364	25.255	10.4	DUBAI INTL
30	40439	38.063	24.144	7.9	YENBO
31	41140	42.586	16.901	6.1	KING ABDULLAH BIN ABDULAZIZ
32	41246	56.65	24.467	2	SOHAR MAJIS
33	41150	50.634	26.271	1.8	BAHRAIN INTL

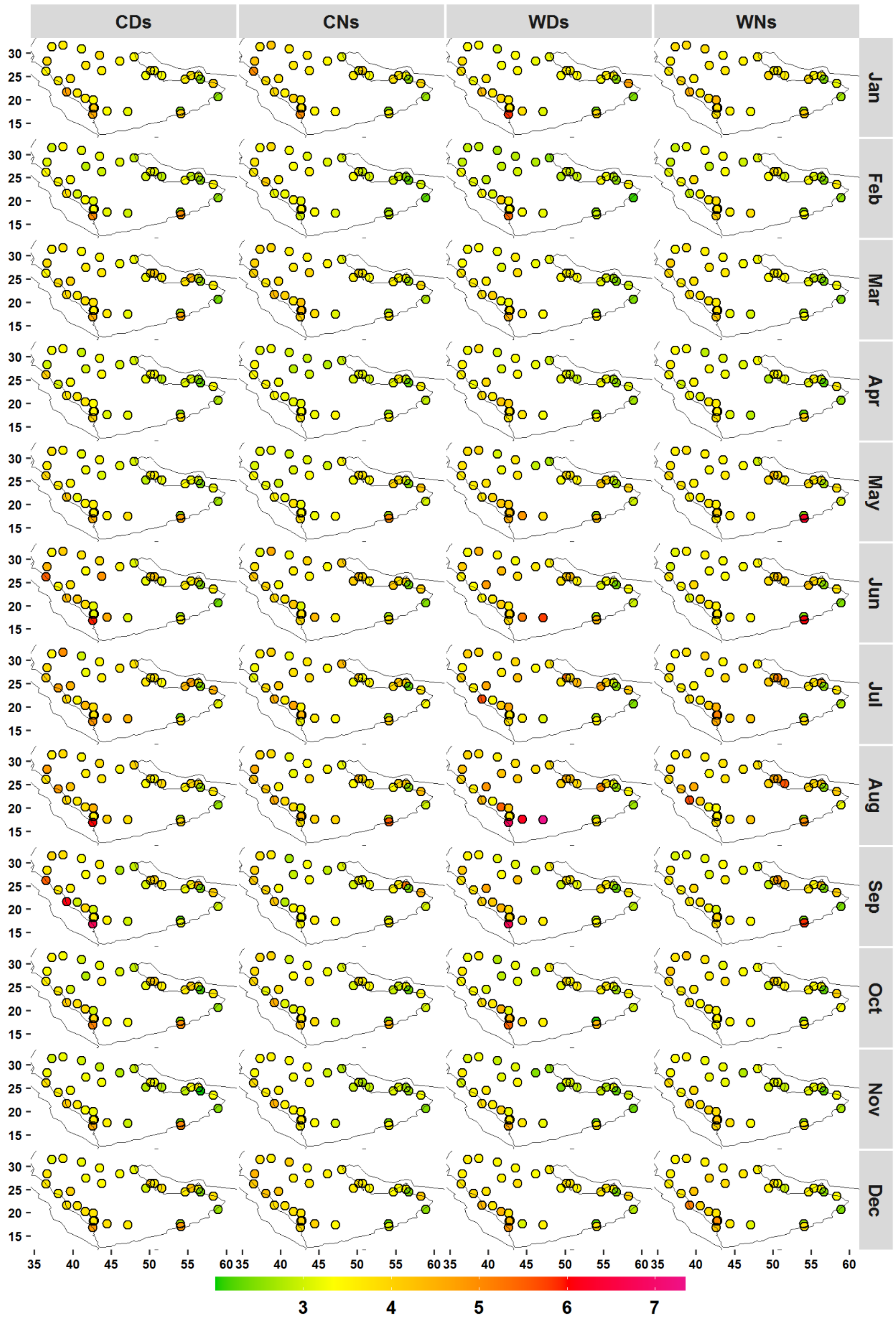

Figure S1. Mean frequency of cold days (CDs), cold nights (CNs), warm days (WDs), and warm nights (WNs) by month.

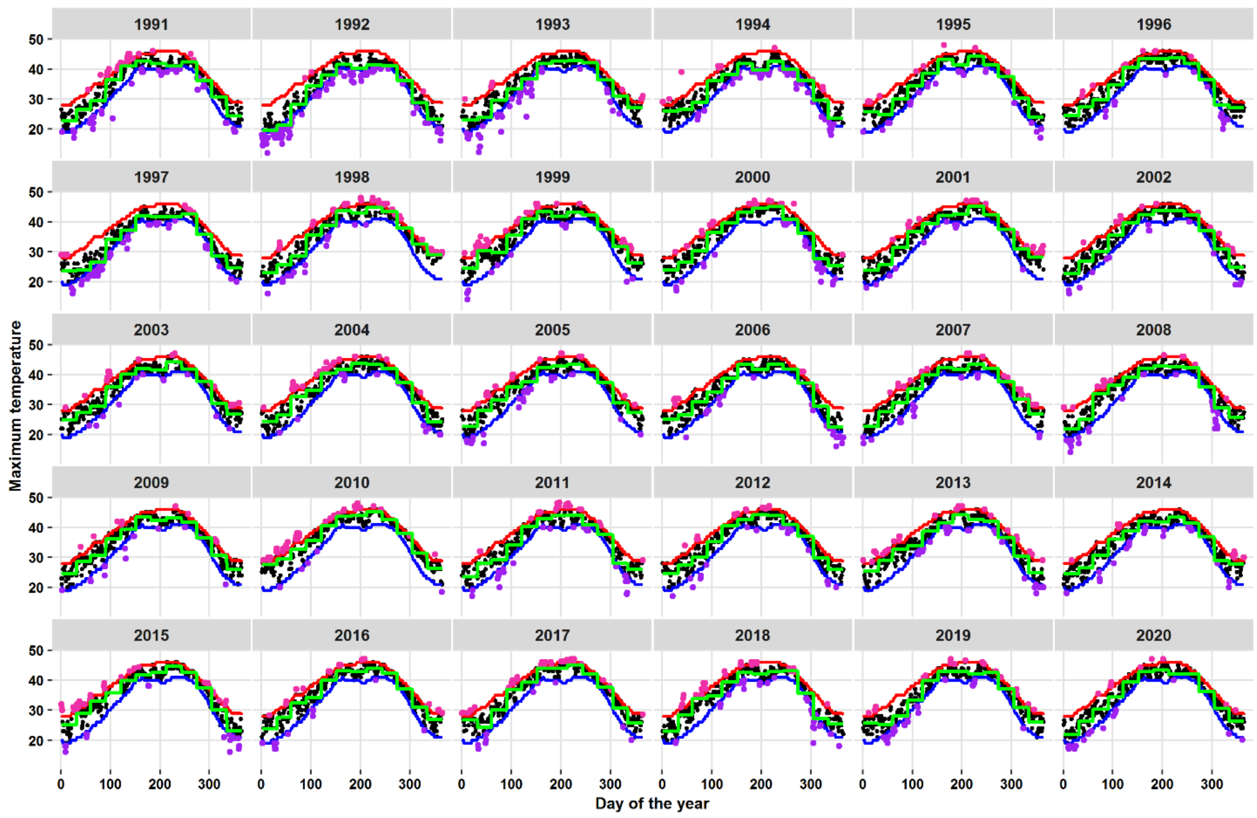

Figure S2. Annual cycles of daily maximum temperature [TX] (black points, °C) at station 40430 along with 90th (red line), 10th (blue line) percentiles, monthly mean TX (green line), detected WDs (red points), and CDs (purple points). Percentiles are estimated from the base period of 1991–2020 on a 15-day centered window.

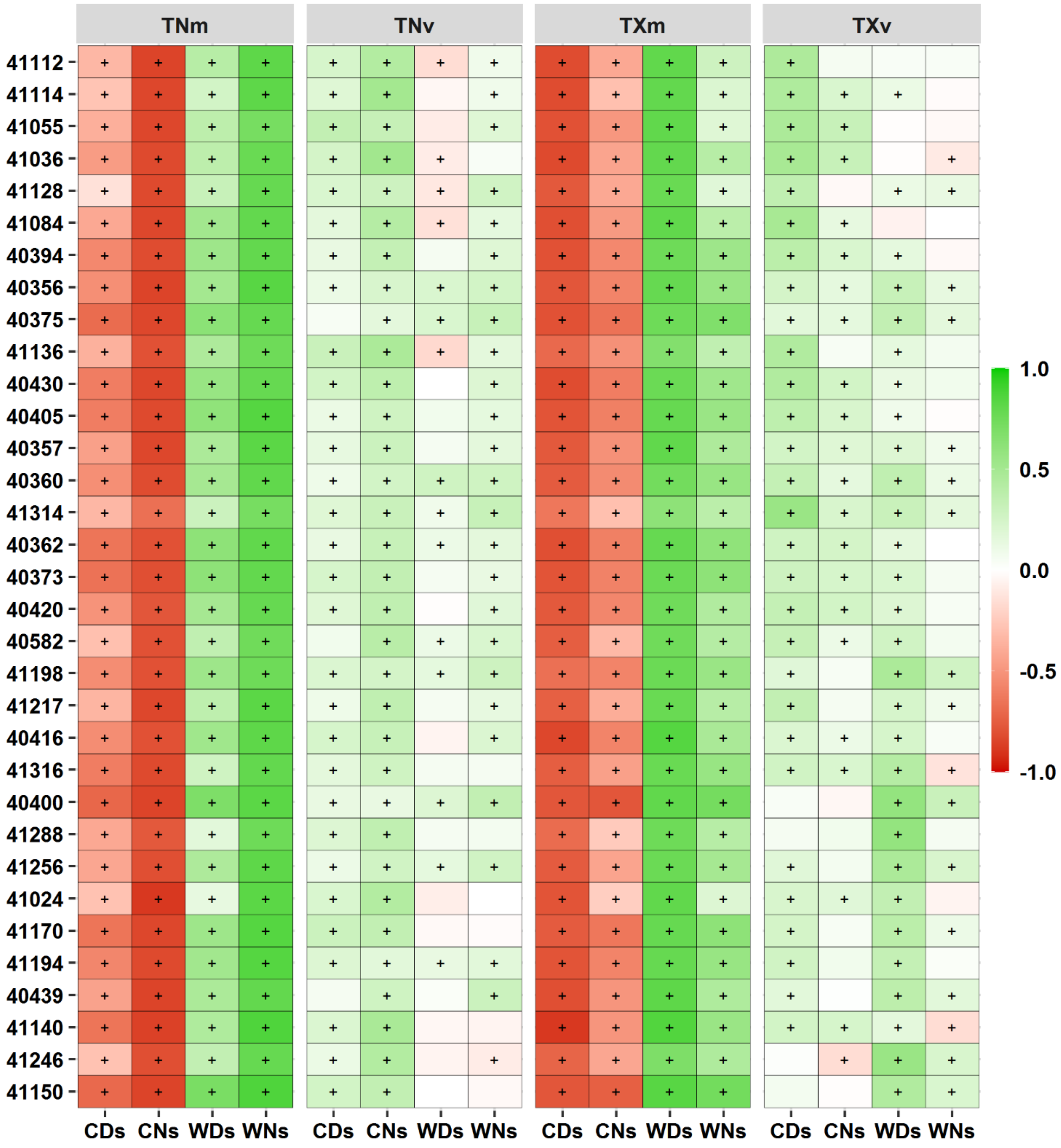

Figure S3. Correlation coefficients (Spearman's rank) between anomalies of mean/variance of TX (TXm/TXv) and TN (TNm/TNv), and frequency of different temperature extreme types. + is statistically significant at the 90% level. All anomalies were obtained with respect to the 1991 to 2020 monthly climatological average. Stations (y-axis) are ordered from high to low elevation.

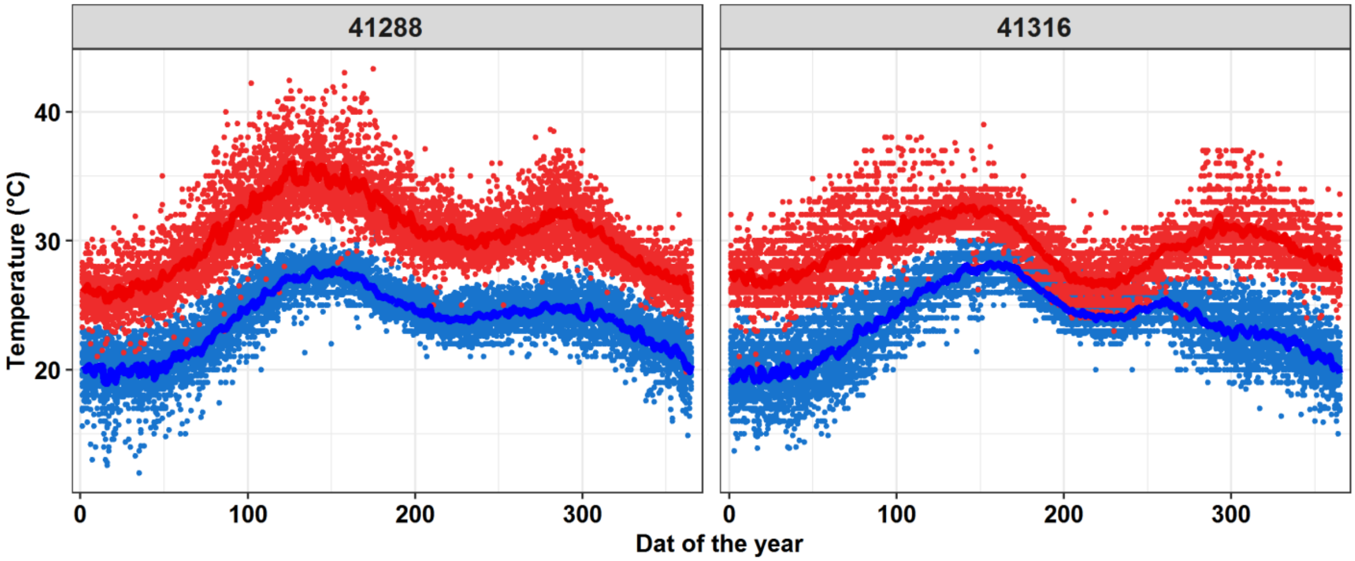

Figure S4. Annual cycles of maximum (red) and minimum (blue) temperatures (1991–2020) at stations 412888 and 41316.

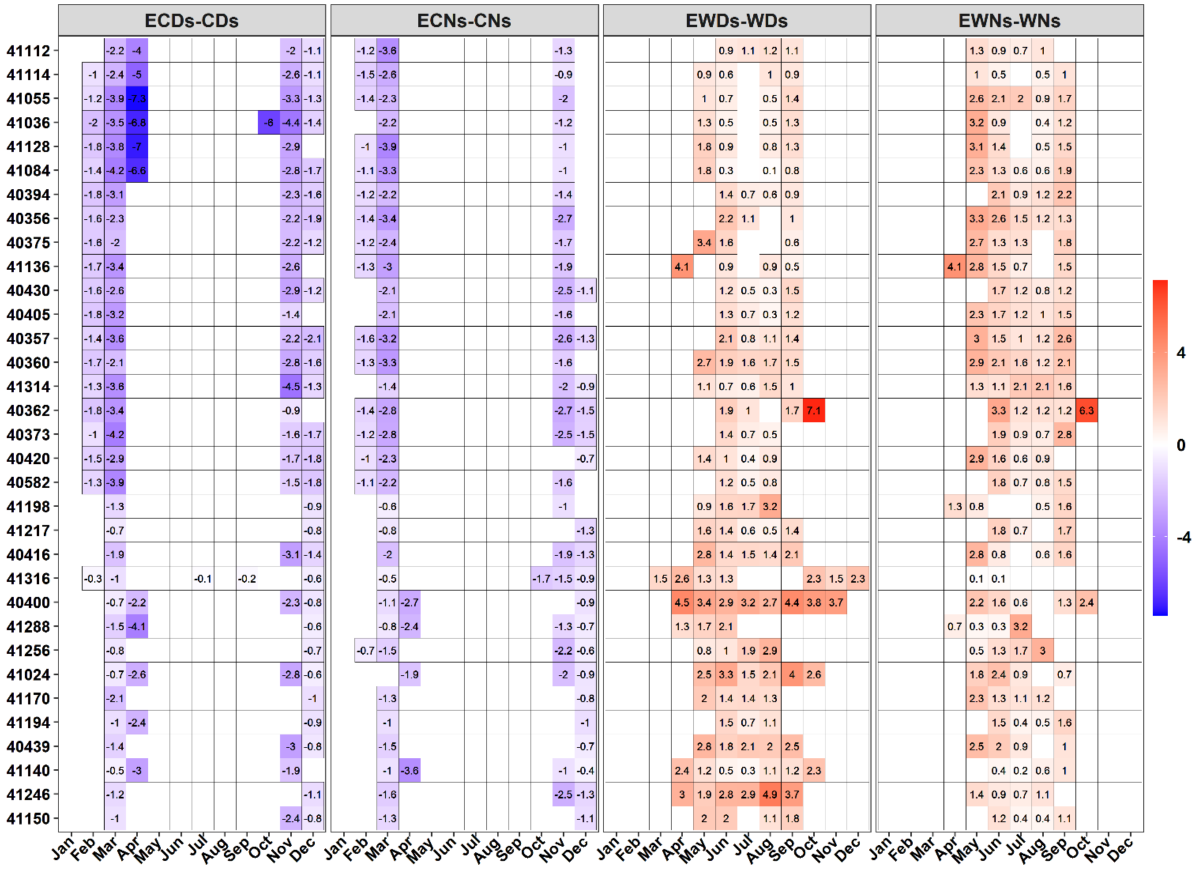

Figure S5. Differences between average relative intensities (°C) of extreme warm/cold days/nights [EWDs/ECDs, ECDs/ECNs] and warm/cold days/nights [WDs/CDs, WNs/CNs] by months. Stations (y-axis) are ordered from high to low elevation.

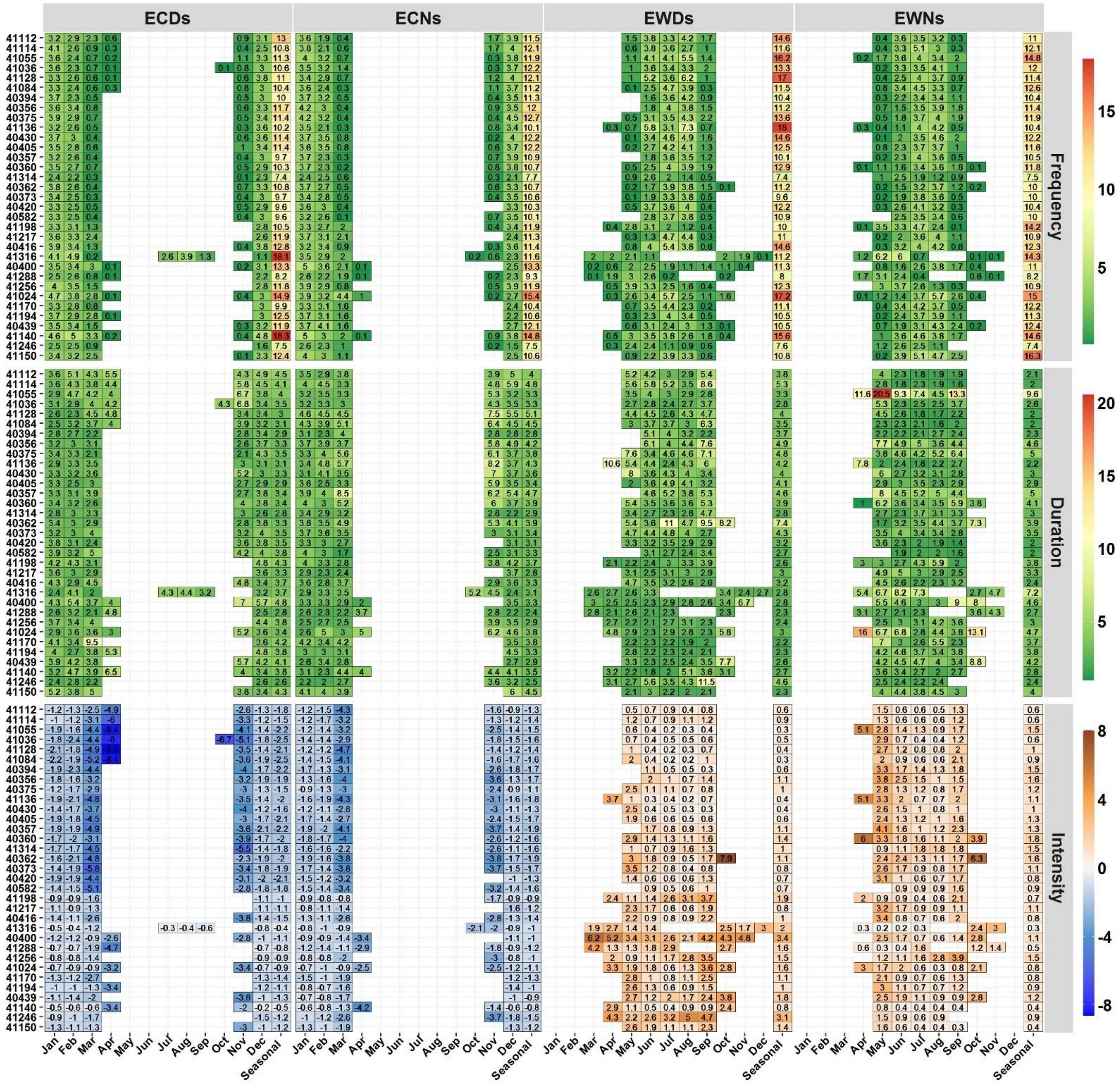

Figure S6. Monthly and seasonal means of frequency, intensity, and duration of extreme warm/cold days and nights along with the corresponding local seasonal averages. Stations (y-axis) are ordered from high to low elevation.

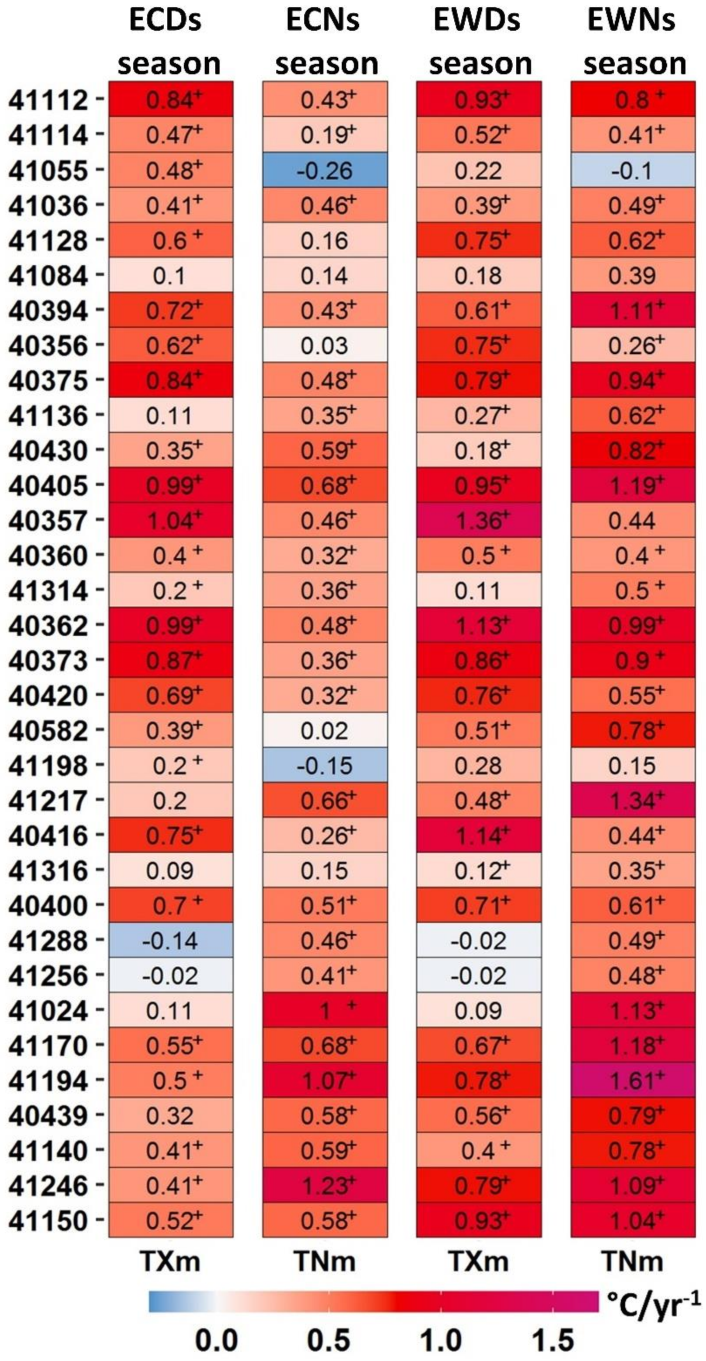

Figure S7. Absolute temporal decadal trends in mean daily maximum (TXm) and minimum (TNm) temperatures at local seasons of each ETEs. + is statistically significant at the 90% level. Stations (y-axis) are ordered from high to low elevation.

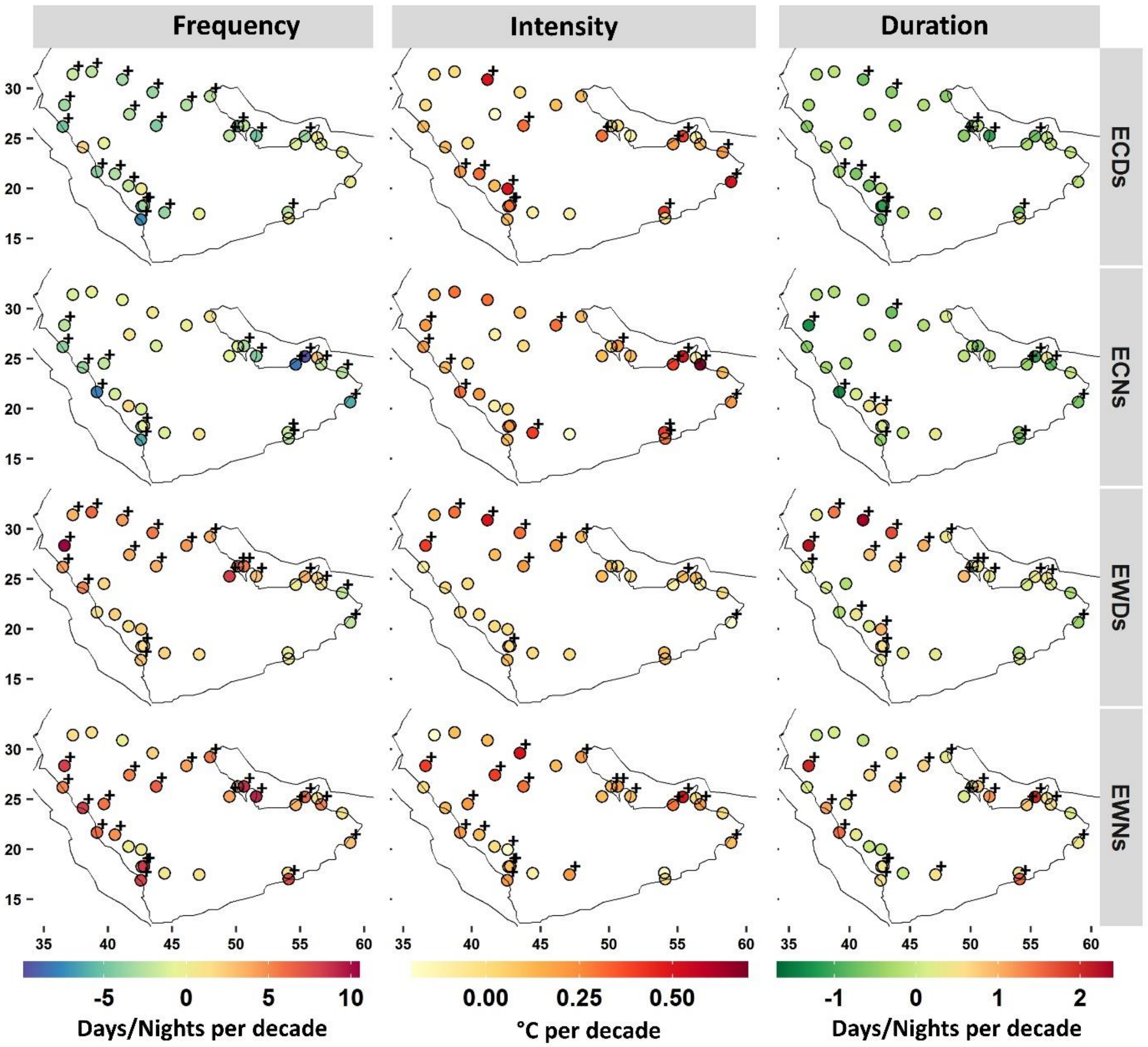

Figure S8. Absolute temporal decadal trends in frequency, duration, and intensity aspects of ETEs during local seasons. + α = 0.10 level of significance.
